# P-1378. Linezolid For Early Syphilis Treatment: Pilot study

**DOI:** 10.1093/ofid/ofae631.1554

**Published:** 2025-01-29

**Authors:** Chrysovalantis Stafylis, Ryan M Nguyen, Kelika Konda, Michael Reyes-Diaz, Kori Keith, Ebony Warren, Byron Johnson, Catherine Creticos, Keith Stokes, Jeffrey Klausner

**Affiliations:** University of Southern California, Los Angeles, California; University of Southern California, Los Angeles, California; Universidad Peruana Cayetano Heredia, Lima, Lima, Peru; Universidad Peruana Cayetano Heredia, Lima, Lima, Peru; University of Southern California Keck School of Medicine, Los Angeles, CA; Howard Brown Health, Chicago, Illinois; Open Arms Healthcare Center, Jackson, Mississippi; Howard Brown Health, Chicago, Illinois; Open Arms Healthcare Center, Jackson, Mississippi; Keck School of Medicine of USC, Los Angeles, California

## Abstract

**Background:**

Syphilis rates have been increasing. At the same time, important shortages of benzathine penicillin G have been reported. Existing alternatives, doxycycline, tetracycline, and azithromycin are inadequate to treat syphilis in all patients due to the absence of safety data in pregnancy and children, or antibiotic resistance, respectively. Linezolid is an FDA-approved, safe, low-cost, oral antibiotic that crosses the blood-brain barrier. It has shown efficacy against Treponema pallidum *in vitro* and in animal studies. Although a recent clinical study evaluating of 600mg Linezolid once daily for 5 days showed limited success, pharmacological simulations show that a longer regimen may be efficacious. We aimed to assess the efficacy of linezolid 600mg, twice a day for 10 days for the treatment of early syphilis.

**Methods:**

We are conducting a randomized, non-comparative, pilot study enrolling 24 adult patients (18 linezolid, 6 penicillin) with primary, secondary, or early latent syphilis in Chicago, Illinois, and Jackson, Mississippi. The experimental arm receives oral linezolid 600mg twice daily for ten days, while the control arm receives benzathine penicillin G 2.4 million units intramuscularly once. The main outcome is a ≥4-fold RPR titer decline by 6 months after treatment. Linezolid participants are monitored for adherence and adverse events during treatment. Clinical and serological response is evaluated at 1-, 3-, and 6-month follow-up visits.

**Results:**

Currently, six out of 24 participants are enrolled in the study; four in the linezolid arm, and two in the penicillin arm. All participants had early latent syphilis. All participants receiving linezolid adhered 100%. One penicillin participant (enrollment RPR 1:16, 3-month RPR 1:2) and one linezolid participant (enrollment RPR 1:64, 3-month RPR 1:16) have completed their 3-month follow-up. No serious adverse reactions have been reported.

**Conclusion:**

Studies of new treatment options for syphilis are underway

**Disclosures:**

**Jeffrey Klausner, MD MPH**, Direct Diagnostics: Advisor/Consultant

